# Special Issue: Beneficial Properties and Safety of Lactic Acid Bacteria

**DOI:** 10.3390/microorganisms11040871

**Published:** 2023-03-28

**Authors:** Wilhelm Heinrich Holzapfel, Svetoslav Dimitrov Todorov

**Affiliations:** 1 Human Effective Microbes Laboratory, Department of Advanced Convergence, Handong Global University, Pohang 37554, Republic of Korea; wilhelm@woodapple.net; 2 ProBacLab, Department of Advanced Convergence, Handong Global University, Pohang 37554, Republic of Korea; 3 ProBacLab, Laboratório de Microbiologia de Alimentos, Departamento de Alimentos e Nutrição Experimental, Faculdade de Ciências Farmacêuticas, Universidade de São Paulo, Sao Paulo 05508-000, SP, Brazil; 4 Food Research Center (FoRC), Departamento de Alimentos e Nutrição Experimental, Faculdade de Ciências Farmacêuticas, Universidade de São Paulo, São Paulo 05508-000, SP, Brazil

The application of LAB in various sectors, including in the biotechnical and food industry, in human and veterinary practice, and in health-promoting practices and cosmetics, has been the subject of intensive research across the globe, with a range of traditional and innovative methods currently being explored. The rediscovery of old practices, the establishment of new processes based on the production and application of different metabolites produced by LAB, and the formation of novel perspectives on the fermentation processes initiated by LAB, have become areas of significant interest in recent years. Various antimicrobial peptides, including bacteriocins, have been proposed as alternatives to antibiotics or have been suggested for use as their synergistic “partners”. The application field of probiotics is being widened to encompass new innovative areas that are targeted towards personalized practice, with the aim of improving human health. An increasingly extensive understanding of bioactive peptides has heralded their application in practices that are alternative or complementary to Western medicine. Approaches to bio-preservation require fewer chemical preservatives and are, currently, thoroughly explored in food research. The enrichment and fortification of food products with biologically active metabolites, including vitamins, antimicrobials, and immunomodulators, are only some of the research areas that ought to be explored as options for the application of various LAB in the food industry.

The concepts associated with the beneficial properties and safety of LAB have been, and always need to be, jointly explored. Even if several LAB strains have been applied historically as safe and beneficial cultures, various other representatives of LAB have been documented as human and animal pathogens, as phytopathogens, and as also including strains associated with spoilage and deterioration [1]. LAB represent a universe of varied microorganisms, with all of them characterized as Gram-positive, catalase negative, as possessing a common metabolism and as initiating the formation of a similar end product (lactic acid) as a result of carbohydrate fermentation [2]. As a diverse group of microorganisms, they are adapted to various ecosystems and environmental conditions, and can grow at different temperatures and use a variety of carbon sources [1,2]. They are associated with virtually all living forms, from simple eukaryotic organisms and plant material, to the skin and GIT of vertebrates, insects, mollusks, crustaceans, etc. They may be described as either beneficial or as pathogens, but they always possess a clear ecological role in numerous life cycles [2]. Of particular note are species such as *Enterococcus* spp., some of which are unmistakably opportunistic pathogens and, when associated with vancomycin resistance, pose a serious health threat to humans and to animals [3]; these pathogens are typically associated with nosocomial infections [3]. Simultaneously, however, LAB also comprise species that play a beneficial role in the production of various plants, dairy and meat fermented food products [4], or even as probiotics [5]. It has been suggested that enterococci are producers of bacteriocins, some of which can be applied in the control of food-borne and hospital-associated (human and veterinary) pathogens [6]. However, before proposing a strain, even one belonging to a species with a history of safe application, its safety properties must be appropriately evaluated; this is a necessary and essential step that must be completed prior to its application in food fermentation, as a probiotic for human and animals, in human and veterinary medicine, or in agricultural practices. The novel tools utilized in the evaluation of the safety of microbial cultures, including DNA-associated experimental approaches, have become routine in the last two decades. Considering this, the validation of safety, both of new microbial and currently applied cultures, is now considered essential. In addition to “classical” PCR-based approaches, whole genome sequencing and the appropriate analysis of the generated data have become routine in the evaluation of the safety profile of microbial cultures [7,8,9].

In this Special Issue of the journal *Microorganisms*, various papers that focus on the beneficial properties of LAB and their safety profiles are presented. The participation of research teams from Russia, Greece, Korea, Malaysia, Taiwan, and France reflects the global interest in building this research collection.

The safety profiles of strains of enterococci, *Lactiplantibacillus plantarum* and *Weissella cibaria* were assessed in the research of Tsanasidou et al. [7], Jang et al. [8] and Hsu et al. [9]. These strains were shown to have various beneficial features, ranging from their production of bacteriocins, to their favorable probiotic properties. In these three research reports, the primary focus of the authors was on safety, which was considered by all as a major priority. Tsanasidou et al. [7] evaluated various dairy-associated enterococci, with the aim of applying them as starter cultures in cheese production. In essence, these strains ought to possess appropriate technological properties, in addition to the production of bacteriocins. The safety assessment of these strains was based on the production of biogenic amines, of specific enzymes, and on features such as hemolysis, antibiotic resistance and the presence of virulence and antibiotic resistance genes. The *E. faecium* and *E. durans* strains both carried multiple enterocin-associated genes (*ent*A, *ent*B, *ent*P, *bac*31, *cyl*) on a strain-specific basis. Moreover, these strains demonstrated moderate to strong esterase–lipase and aminopeptidase activities, which are considered to be highly beneficial in cheese production. Tsanasidou et al. [7] concluded that, with the exception of *cyl*-positive *E. durans* KE108, most *E. faecium* and *E. durans* strains can be considered as safe and be deemed potentially applicable as adjunct cultures in the production of traditional Greek cheeses. 

Jang et al. [8] evaluated the safety of *Weissella cibaria*; this is a strain isolated from kimchi, a popular Korean fermented food that is associated with several health benefits linked to the bacterial metabolites and various LAB [10]. However, further investigations into the kimchi microbiome revealed that indigenous strains possessed distinct beneficial properties (showing potential as putative probiotics), but also that these strains posed a potential health hazard to consumers. This may be a consequence of the poor hygienic conditions that can arise in small-scale production facilities. The study of Jang et al. [8] provides valuable information on the safety of kimchi and on the isolation of the *W. cibaria* strain JW15, which represents one of the most predominant species involved in the initial fermentation of kimchi. The presence of this strain in the final product indicates its adjunct benefits, which are reflected by kimchi’s immune-modulating, antagonistic and antioxidant properties; this also confirms its safety [8].

*Lb. plantarum* is a microbial species that is isolated from a variety of ecological niches, including numerous fermented food products [11]. Due to their extensive history of applicative use, strains *of* the *Lb. plantarum* species are considered to be generally recognized as safe (GRAS). However, as aforementioned, safety is a strain-based characteristic and thus cannot be associated with an entire species. Hsu et al. [9] reported on *Lb. plantarum* TWK10, isolated from Taiwanese pickled cabbage; in addition to its suggested probiotic properties, as evaluated in mice and humans, the authors assessed this strain’s safety via in vitro and in vivo experiments. In addition to the performance of physiological and biochemical tests, whole genome sequencing and an analysis of the obtained results revealed that *Lb. plantarum* TWK10 can be considered as safe; this is in addition to the fact that no significant increase in the incidence of reverse mutations or chromosomal aberrations was observed. 

The beneficial properties of LAB can cover an extensive list of applications. Generally, the beneficial roles of LAB are analogous with three principal pillars: their health-promoting/probiotic effects, their utilization as starter cultures in various food fermentation processes, and in the production of antimicrobial peptides (bacteriocins) [12,13]. However, the role of LAB is far more extensive than this relatively limited scope of application. In their paper, George et al. [14] propose that some LAB might be applied as effective tools in the reduction of heavy metals. The contamination of animal feed with toxic metals can be transferred to humans via the food chain, presenting a health hazard to both animals and humans. George et al. [14] explored the role of LAB in the adsorption and/or sequestration of heavy metals, and via this, ways in which to reduce their bioavailability and toxic effects in the GIT of consumers. The specific participation of different microbial groups was recorded, with a particular focus on Firmicutes such as LAB (formerly classified as *Lactobacillus* spp., some *Lactococcus*, *Pediococcus*, and *Carnobacterium* representatives), Actinobacteria, and Proteobacteria. 

The beneficial role of LAB might associate with the production of bioactive peptides during the fermentation process of food products, and their role in providing health-promoting properties. Previously, Atanasova et al. [15] reported on the various bioactive peptides recorded in white brine cheeses and their potential role in the control of cardiovascular diseases; such metabolites can be classified as postbiotics [16]. In addition, Kurbanova et al. [17] focused on the identification of various bioactive peptides in maturated cheeses. By performing an in silico analysis, the authors revealed the relationship between the class of applied starter culture and the formation of the bioactivity and other health benefits that characterize peptides in Caciotta-type cheeses; the studied semi-hard cheese was described as with the presence of an angiotensin-converting enzymes (ACE) inhibitor and an dipeptidyl peptidase IV (DPP-4) inhibitor.

The ameliorating effects of the application of *Lb. plantarum* DR7 on of the soreness symptoms and frequency of upper respiratory tract infections (URTIs) was evaluated in a study of Altadill et al. [18]; this study included 109 adults voluntiers and an evaluation of their symptoms after a 12-week consumption of 10^9^ CFU/day *Lb. plantarum* DR7. The application of *Lb. plantarum* DR7 had an explicitly positive effect on the symptoms of URTIs and the fever experienced by the participants in the controlled study. Altadill et al. [18] suggested that *Lb. plantarum* DR7 could be considered as an effective probiotic in the reduction of URTI symptoms. These results must be regarded as promising observations and key to the selection and evaluation of possible probiotics in the prevention and/or treatment of COVID-19-associated symptoms. 

*Ligilactobacillus salivarius* MG242, *Limosilactobacillus fermentum* MG901 and *Lb. plantarum* MG989 were suggested as putative probiotics for the control of bacterial vaginosis; their beneficial effects were evaluated in a mouse model [19]. The oral administration of these strains revealed that *Gardnerella vaginalis* was inhibited by up to 43% and that the expression of the pro-inflammatory cytokines IL-1β, TNF-α and myeloperoxidase was downregulated [19]. The safety evaluation of these strains regarding hemolytic activity, antibiotic susceptibility, enzyme activity and lactic acid production revealed that all could be considered as promising candidates for the oral administration of probiotics, with particular benefits being directed at the control of bacterial vaginosis.

In last two decades, the link between the GIT and various organs of the human body and other vertebrates has been extensively explored in several research projects [20]. Lee et al. [21] proposed the beneficial influence of orally administering probiotics to the liver. Liver damage is not only engendered by alcohol abuse, but various diseases can also lead to the malfunction of the liver; indeed, it may also be damaged by the side effects of various drugs and intoxicating agents. An imbalance in the intestinal microbiota may result from excessive and chronic alcohol consumption and thus lead to severe liver diseases, thereby inducing the excessive release of endotoxins in the hepatic portal vein [21]. Lee et al. [21] reported that strains of the species *Levilactobacillus brevis*, *Limosilactobacillus reuteri*, and *Lb. fermentum* may exert a protective effect on ethanol-induced HepG2 cells via increased aldehyde dehydrogenase (ALDH) levels, downregulated lipid peroxidation and liver transferase in the ethanol-induced HepG2 cells. The safety of the strains *Lb. brevis* MG5280 and MG5311, *Lb. reuteri* MG5458, and *Lb. fermentum* MG4237 and MG4294 was also evaluated in view of their potential application as probiotics, in addition to their stability and adhesion capabilities in the GIT.

The traditional applications of bacteriocins in bio-preservation are being widened by current research projects that suggests that bacteriocins might be utilized as tools in the control of relevant clinical pathogens and in multidrug-resistant strains [13]. Fugaban et al. [4] reported on the production of bacteriocin by the *E. faecium* strains (ST651ea, ST7119ea and ST7319ea) found in traditional Korean fermented soybean pastes and their activity against vancomycin-resistant enterococci and *Listeria monocytogenes* strains. These bacteriocins were studied by utilizing the appropriate biochemical, physiological, and biomolecular approaches. PCR-based analysis showed that these strains can carry more than one gene associated with bacteriocin production. The authors suggest that these strains could potentially be utilized in biocontrol in the food and feed industries, and also in the reduction in and/or elimination of vancomycin-resistant enterococci-related infections in both veterinary and clinical settings.

Most of the commercialized probiotics on the market are associated either with fermented dairy products, other food commodities or with lyophilized preparations. Arellano-Ayala et al. [22] explored probiotic efficacy from a different perspective, by focusing on the stability and viability of bacterial cells after lyophilization and dehydration. It is thought that both the direct consumption of lyophilized probiotics or their reconstitution before application could play a critical role in their viability and functionality, especially under stress conditions in the upper GIT. After preliminary screening, specific rehydration formulations were prepared and evaluated by checking the survival and functionality of rehydrated *Lb. plantarum* HAC03. An optimal rehydration combination, consisting of non-protein nitrogen compounds, sugars, and B-active formulation, was further evaluated for its protective impact on *Lb. plantarum* HAC03 and on survival in the gastrointestinal model. Rehydration with the B-active formulation resulted in enhanced viability and survival after passing simulated gastro-intestinal stress conditions; it also positively influenced adhesion to the Caco-2 cells of *Lb. plantarum* HAC03 by changing the bacterial surface charge, which was evaluated as zeta potential. 

LAB are one of the more frequently explored microbial groups, together with bifidobacteria, in probiotic and bio-preservation research and application. From “simple” starter cultures to “sophisticated” bioprotective cultures and “powerful” probiotics, various LAB have been revealed to play numerous beneficial roles. Well-founded microbiological studies comprise a fundamental basis for further applications of LAB. As part of the researcher’s ethical responsibilities, the appropriate safety evaluation and correct taxonomic identification should be conducted for any microbial culture intended to be applied in food fermentation, as a probiotic, or as a producer of postbiotics for human and other vertebrates. Performing the appropriate biochemical, physiological and biomolecular tests is a vital aspect of the evaluation of LAB’s beneficial properties. Whole genome sequencing, in conjunction with bioinformatic analysis, has become a routine step in the evaluation of the safety profile and beneficial properties of putatively beneficial LAB strains. Over 2000 years ago, Hippocrates dreamt that food would be our medicine, and that medicine would be our food. We are, indeed, approaching the realization of that dream by implementing beneficial LAB. 

We believe that this collection of articles, published as a Special Issue in the journal *Microorganisms* on the beneficial properties and safety of LAB, will enrich readers’ knowledge on the application of LAB and will inspire personal research projects. 
